# Anaesthetic management of an abdominal aortic aneurysmorrhaphy in Klippel-Trenaunay-Weber syndrome: a case report

**DOI:** 10.1186/s12871-022-01761-y

**Published:** 2022-07-11

**Authors:** Yuichi Tanaka, Shun-Ichiro Sakamoto, Hiroyasu Bito, Atsuhiro Sakamoto

**Affiliations:** 1 Department of Anesthesiology, Musashikosugi Hospital, Nippon Medical School, 1-383, Kosugicho, Nakahara-ku, 211-8533 Kawasaki, Kanagawa Japan; 2 Department of Cardiovascular Surgery, Musashikosugi Hospital, Nippon Medical School, 1-383, Kosugicho, Nakahara-ku, 211-8533 Kawasaki, Kanagawa Japan; 3 Department of Anesthesiology, Nippon Medical School, 1-1-5, Sendagi, Bunkyo-ku, 113-8603 Tokyo, Japan

**Keywords:** Klippel-Trenaunay-Weber syndrome, Abdominal aortic aneurysm, Anaesthesia

## Abstract

**Background:**

Klippel-Trenaunay-Weber syndrome (KTWS) is a rare congenital malformation. Although there have been few reports on anaesthetic management of patients with KTWS, there is a lack of data on anaesthetic management for abdominal aortic aneurysm (AAA) surgeries in these patients.

**Case presentation:**

A 74-year-old man (height, 160 cm and body weight, 51.5 kg) with KTWS was scheduled for AAA replacement. Abdominal computed tomography (CT) showed prominent tortuosity below the abdominal aorta with an infrarenal abdominal aortic aneurysm, right common iliac artery aneurysm, and right external iliac artery aneurysm. Moreover, a remarkably noted arteriovenous fistula had developed between the aneurysm and peripheral artery. General anaesthesia was induced. Furthermore, a central venous catheter and an 8.5 French sheath in the left internal jugular vein were inserted. During the operation, bleeding from a collateral vessel in the cross-clamped aorta led the surgeon to decide to perform aneurysmorrhaphy. Intraoperatively, blood loss was 1500 ml, and 20 units of red blood cell concentrate were used.

**Conclusions:**

Regarding AAA procedures in patients with KTWS, aortic cross-clamping may not sufficiently intercept blood flow due to collateral vessels. In these patients, the anaesthesiologist must be prepared to transfuse blood more rapidly and frequently than during normal AAA procedures.

## Background

Klippel-Trenaunay-Weber syndrome (KTWS) is a rare congenital malformation characterised by a triad of unilateral flat red haemangiomas, hypertrophy of bone or soft tissues, and varicose veins [1]. It is estimated that only one in 27,500 live births has this disorder [2].

Few reports are available on anaesthetic management of patients with KTWS [2–4]. In addition, most of these reports described plastic and orthopaedic surgeries, such as lower limb amputation. There have been no reports on anaesthesia management for abdominal aortic aneurysm (AAA) surgeries in patients with KTWS. We report, herein, our experience with performing anaesthesia management for AAA suture.

## Case presentation

**Fig. 1:**
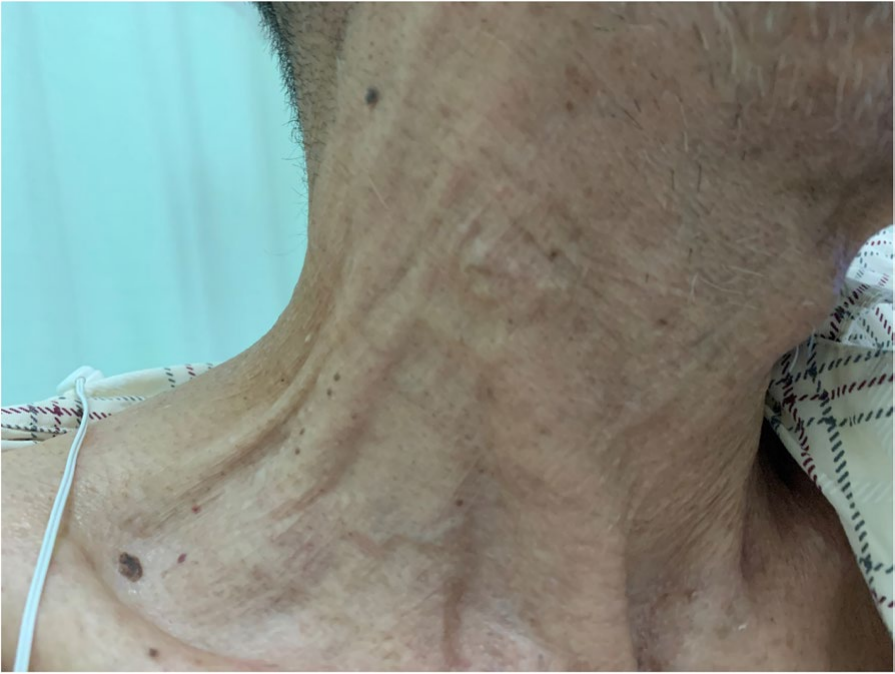
Development and tortuosity of superficial vessels in the right neck

**Fig. 2:**
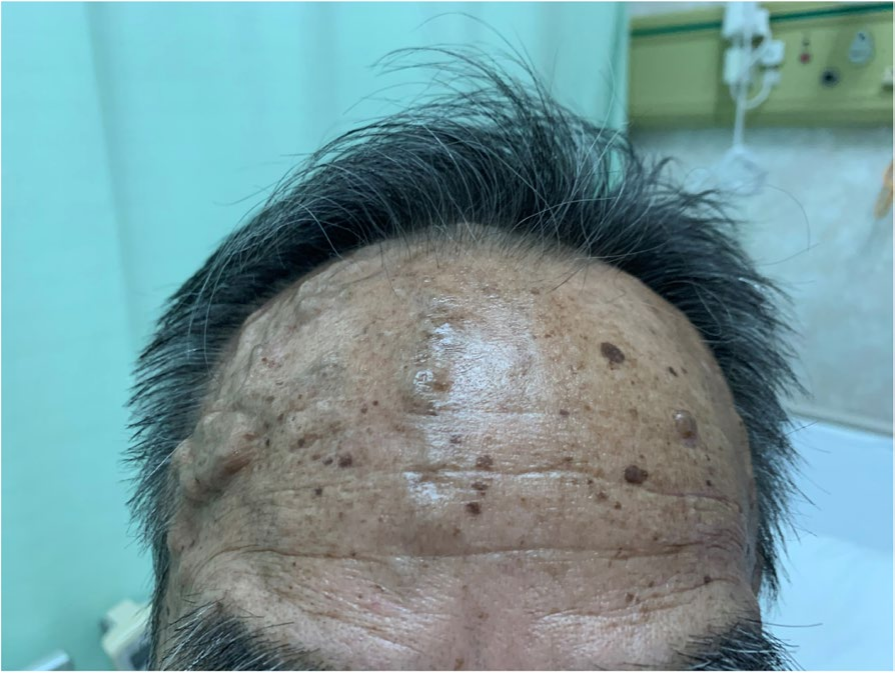
Development and tortuosity of superficial vessels in the right forehead

**Fig. 3:**
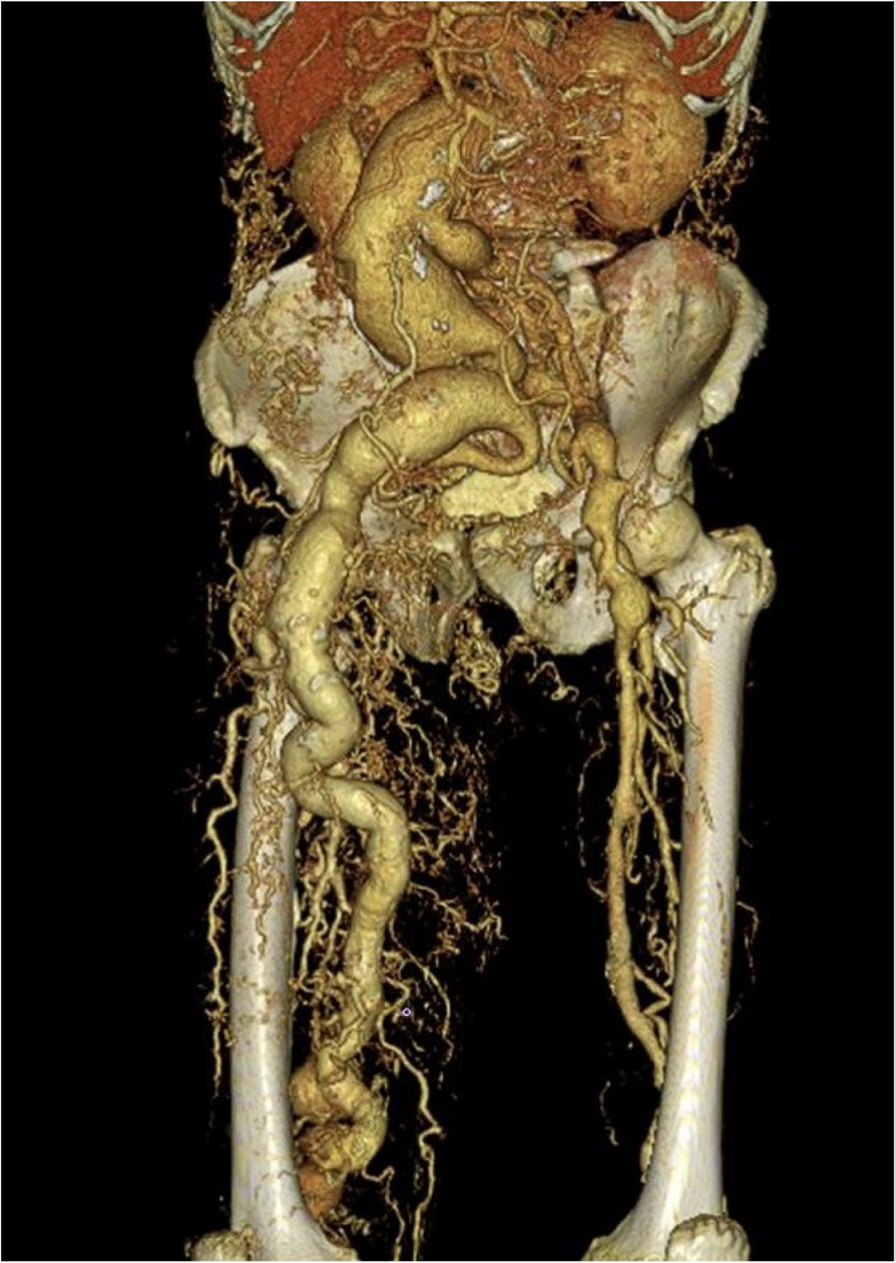
Preoperative abdominal three-dimensional computed tomography

A 74-year-old man (height, 160 cm and body weight, 51.5 kg) had swelling and pain in the right leg. The patient had been diagnosed with KTWS with arteriovenous fistula in the right leg 10 years prior to presentation to our clinic. He had undergone an open total gastrectomy for gastric cancer three years previously. In the past year, the patient’s abdominal aorta became enlarged to a size indicated for surgery. Furthermore, the patient complained of continuous pain in the abdominal region suggestive of an aortic aneurysm with a maximum diameter. Since the patient had a history of abdominal surgery, endovascular aortic repair was considered. However, this repair option was considered inappropriate due to poor vascular characteristics in this patient. We decided to re-open the abdomen for vascular replacement of the abdominal aorta. On physical examination, in addition to swelling of the right leg, significant development and tortuosity of superficial vessels in the right neck and right forehead were observed (Figs. 1 and 2). Vessels in the upper limbs, left lower limb, left neck, and left forehead were somewhat tortuous but otherwise generally normal. Abdominal computed tomography (CT) showed prominent tortuosity below the abdominal aorta with a maximum short diameter of a 60-mm infrarenal abdominal aortic aneurysm, 55-mm right common iliac artery aneurysm, and 42-mm right external iliac artery aneurysm. In addition, a remarkably observed arteriovenous fistula had developed between the aneurysm and peripheral artery, which had dilated towards the popliteal artery. Additionally, the distal popliteal artery was occluded (Fig. 3). Preoperative laboratory test results showed low levels of haemoglobin (9.7 g/dL) and cholinesterase (109 units/L). No coagulation abnormalities were detected. Erythrocyte transfusion was not performed preoperatively. A preoperative electrocardiogram showed atrial fibrillation. Transthoracic echocardiography showed enlargement of the right atrium and ventricle with moderate tricuspid regurgitation and mild mitral regurgitation due to an arteriovenous fistula in the right leg. The left ventricular ejection fraction was 57%. The patient’s ejection fraction decreased. However, since the patient had no problems with daily activities or signs of heart failure, we considered his preoperative haemodynamics to be stable. No obvious cerebrovascular malformations were noted on preoperative examination. Based on these findings, we have considered various solutions to resolve the anticipated problems during anaesthesia management (Table 1).

**Table 1:** Anaesthetic considerations for the KTWS patients

Problems	Solutions
Risk of vascular puncture due to well-developed body surface vessels	Use echo during the nerve block
High cardiac output heart failure due to Arteriovenous (AV) shunts and associated valvular disease	Circulatory management for each cardiac disease
Risk of inadequate clamping and hemorrhage due to development of collaterals	Ensure sufficient blood products and the large transfusion catheters
Development of abnormal vessels in the epidural space	Preoperative Computed tomography (CT) scan, avoid epidural puncture

General anaesthesia was induced with fentanyl (250 µg), propofol (80 mg), and rocuronium bromide (50 mg) and was maintained with sevoflurane, fentanyl, and rocuronium bromide. An arterial catheter was placed in the left radial artery, whereas a central venous catheter and an 8.5 French sheath were placed in the left internal jugular vein. We used Flotrac™ sensor (Edwards Lifesciences Corp, Irvine, CA, USA). A regional cerebral oxygen saturation (rSO_2_) monitor was placed on the left forehead. Surgery was planned for graft replacement or aneurysmorrhaphy, which would be selected according to the amount of bleeding after cross-clamping of aorta. It was considered that graft replacement could potentially influence the collateral circulation via arteriovenous fistula, resulting in venous thrombosis or limb ischemia. After aortic cross-clamping, incision of the aorta triggered excessive bleeding from the collateral vessel in the aneurysm. As a result, the surgeon changed tactics and decided to perform aneurysmorrhaphy. The aneurysm was decompressed with longitudinal aortotomy to reduce its diameter. The mattress suture was made underneath the side clamp, and then reinforced by a running suture using a Teflon felt on each side. Following the operation, since the right side had markedly developed superficial vessels and there were concerns about the risk of vascular injury, we performed a posterior transversus abdominis plane block (TAPB) on the left side only. The operative time was 274 min, anaesthesia time was 387 min, and blood loss was 1500 ml. Intraoperatively, 20 units of red blood cell concentrate, 8 units of fresh frozen plasma, and 20 units of platelets were used. Cardiac index generally remained at 2–2.5 L/min/m^2^, while the rSO_2_ value generally remained stable at around 60 throughout the operation. The patient was extubated in the operating room, discharged from the intensive care unit on the second sick day, and discharged from the hospital on the thirteenth sick day. The patient’s pain was under control in the intensive care unit after the operation.

## Discussion and conclusions

KTWS is a rare disease. There have been a few surgical publications, including one by Servelle describing 768 cases [4]. However, the majority of the cases described in that report involved cutaneous haemangiomas. In cases of haemangiomas of the body surface, KTWS malformations have been reported to persist or worsen in many surgically treated patients (approximately 90%). Therefore, conservative treatment options, such as physiotherapy, compression therapy, and wound care were recommended [5]. When surgical procedures are necessary, less-invasive techniques, such as laser coagulation and sclerotherapy, can be frequently used [5].

In cases similar to the one described here, the surgical team must consider surgical indications for abdominal aortic aneurysms. In general, when abdominal aortic aneurysm grows to a size greater than 55 mm, clinicians recommend considering elective repair in men [6]. However, there is no evidence of a threshold in patients with KTWS. We decided to perform open surgery in the patient because the maximum short diameter was 55 mm, and he had abdominal symptoms.

Surgeons must conduct a systemic search for presence of capillary and venous malformations; varicose veins; and arteriovenous fistulas. A chest CT scan may also be useful for determining presence of pulmonary arteriovenous fistulas. Since arteriovenous fistulas can lead to high cardiac output and heart failure, we should carefully interrogate and document any history of heart failure and perform preoperative transthoracic echocardiography as part of surgical planning to better visualise the anatomy prior to opening. Regarding large varicose veins presence on the extremities or trunk of the body, chronic blood coagulation abnormalities persist, and high D-dimer and low fibrinogen levels might be noted. In addition, bleeding or surgical invasion may lead to disseminated intravascular coagulation. Patients with KTWS are susceptible to varicose vein formation, which can lead to thrombophilia. This in turn could lead to chronic disseminated intravascular coagulation and Kasabach-Merritt syndrome [5]. Therefore, in anaesthetising patients with KTWS for AAA surgery, it is essential to perform angiography and determine the preoperative coagulation status, which are key to determining the need for surgery and the suitable surgical procedure.

Few reports on anaesthetic management of patients with KTWS have been published in the medical literature. Both general anaesthesia and different techniques for regional anaesthesia have been used [2]. Considering epidural puncture, since haemangiomas and arteriovenous malformations can complicate the epidural space, even in absence of abnormal neurological findings, the anaesthesiologists should examine patient preoperatively for presence of abnormal blood vessels in the epidural space [2, 7, 8]. Given the risk of serious adverse events due to presence of unexpected vascular malformations, it is strongly recommended that spinal cord imaging be performed prior to procedure when epidural or spinal subarachnoid block is used [5].

In patients with KTWS, vascular malformations and coagulopathy inherent to this disorder might increase the risk of bleeding during some surgeries. According to Barbara et al. [5], although tourniquets were used in 50% of their patients, the use of tourniquets did not eliminate the possibility of significant or life-threatening surgical bleeding. The need for both adequate venous access and blood products is paramount in surgical management in patients with KTWS with potentials for massive bleeding.

We chose to use only general anaesthesia in this patient due to reports of complications due to haemangiomas and arteriovenous malformations in the epidural space. Since the patient’s vascular lesions were systemic, and massive intraoperative bleeding was expected, we considered a large venous catheter to be essential. Due to developing superficial vessels on the right side, the rSO_2_ monitor could only be applied to the left side. Pereda et al. suggested that patients with KTWS may experience difficult intubation due to soft tissue hypertrophy, haemangiomas of the upper airway, and facial deformities [2]. However, Barbara et al. indicated that difficult intubation was not frequent in clinical practice [5]. In general cases, it is possible to predict intubation difficulties based on physical findings, as well as head and neck CT. In the present case, intubation was easily performed.

Preoperative angiography showed an aneurysm with extensive collateral circulation (Fig. 3). When the aneurysm was opened, excessive bleeding was noted, impeding the identification of the collateral vessels. As a result, the operation was changed to aneurysmorrhaphy. Unfortunately, the autologous blood collection device failed and could not be used during the operation. Therefore, blood transfusion was required. Due to high risk of bleeding in such cases, the use of an intraoperative autologous blood collection device may help reduce the amount of blood needed for transfusion, if possible.

We attempted to perform bilateral TAPB postoperatively. However, superficial echography showed many collateral vessels on the right side of the abdominal surface. Therefore, TAPB was performed only on the left side. Superficial echography should always be used in patients with KTWS when performing nerve blocks to reduce risks of vascular puncture.

In conclusion, we report the safe anaesthetic management of a patient with KTWS undergoing abdominal aortic aneurysmorrhaphy. In patients with KTWS, it is highly likely that aortic cross-clamping might not sufficiently block blood flow due to abundance of abnormal collateral vessels. Therefore, as in the present case, massive bleeding after aortic cross-clamping may occur, requiring more rapid and frequent blood transfusions than in a normal AAA procedure. Anaesthesiologists should manage anaesthesia with this risk in mind.

## Data Availability

Not applicable.

## Abbreviations

AAA: Abdominal aortic aneurysm
CT: Computed tomography
KTWS: Klippel-Trenaunay-Weber syndrome
rSO_2_: Regional cerebral oxygen saturation
TAPB: Transversus abdominis plane block
